# Mepolizumab in Hypereosinophilic Syndrome: A Systematic Review and Meta-analysis

**DOI:** 10.6061/clinics/2021/e3271

**Published:** 2021-09-28

**Authors:** José Mario Alves, Francisco Eduardo Prota, Danilo Villagelin, Fernanda Bley, Wanderley Marques Bernardo

**Affiliations:** I Unimed Campinas Cooperativa de Trabalho Medico - Gestao e Valor em Saude, Campinas, SP, BR.; II Ebenezer Gestao em Saude - Medicina Baseada em Evidencia, Sao Paulo, SP, BR.; III Unimed Fesp - Federacao das Unimeds do Estado de Sao Paulo, Sao Paulo, SP, BR.; IV Faculdade de Medicina FMUSP, Universidade de Sao Paulo, Sao Paulo, SP, BR.

**Keywords:** Mepolizumab, Humanized Monoclonal Antibody, Hypereosinophilic Syndrome

## Abstract

We aimed to evaluate the efficacy and safety of mepolizumab (MEP) in the management of hypereosinophilic syndrome (HES). A systematic search was performed, and articles published until March 2021 were analyzed. The primary efficacy results evaluated were hospitalization rate related to HES, morbidity (new or worsening), relapses/failure, treatment-related adverse effects, prednisone dosage ≤10 mg/day for ≥8 weeks, and eosinophil count <600/μL for ≥8 weeks. A meta-analysis was conducted, when appropriate. Three randomized controlled trials (RCTs), with a total of 255 patients, were included. The studies contemplated the use of MEP 300 mg/SC or 750 mg/IV. According to the evaluation of the proposed outcomes, when relapse rates/therapeutic failures were assessed, there was a 26% reduction with MEP 300 mg/SC (RD=-0.26; 95% CI: -0.44 to -0.08; *p*=0.04) and 48% reduction with MEP 750 mg/IV (RD=-0.48; 95% CI: -0.67, -0.30; *p*<0.00001). For the outcomes, prednisone dosage ≤10 mg/day for ≥8 weeks was 48% (RD=0.48; 95% CI: 0.35 to 0.62; *p*<0.00001), and the eosinophil count <600/μL for ≥8 weeks was 51% (RD=0.51; 95% CI: 0.38 to 0.63; *p*<0.00001), both showed a reduction with MEP 300 mg/IV and 750 mg/IV. No statistically significant differences in treatment-related adverse effects outcomes were observed for either dosage (RD=0.09; 95% CI: -0.05 to 0.24; *p*=0.20; RD=0.09; 95% CI: -0.11 to 0.29; *p*=0.39). Despite the positive effects observed for the studied outcomes, the exact significance remains unclear.

## INTRODUCTION

Hypereosinophilic syndrome (HES) is a group of disorders marked by the sustained overproduction of eosinophils, in which eosinophilic infiltration and the release of mediators cause damage to multiple organs, including the skin, cardiopulmonary region, and the gastrointestinal tract. HES is rare, and its true prevalence is unknown. In a study that used the clinical code of eosinophilia to identify patients with HES in the Surveillance, Epidemiology, and Final Results (SEER) database, the estimated prevalence was 0.36-6.3 per 100,000 individuals. Most patients are between 20 and 50 years of age at the time of diagnosis, although this condition can also be observed in children (1).

Eosinophilia can be considered mild, when the absolute eosinophil count (AEC) in peripheral blood is above the reference limit (AEC<1.500/mm^3^), moderate (AEC between 1.500 and 5.000/mm^3^), and severe (AEC>5.000/mm^3^). Hypereosinophilia (HE) occurs when there is a moderate to severe increase in AEC (>1.500/mm^3^ or >1.500 cells/µL) in two separate tests within at least one month and/or pathological confirmation of HE tissue (1-4). HES is characterized by the association of HE (as defined above) with damage and/or dysfunction mediated by eosinophils in organs, as long as other potential causes of the damage have been excluded (1).

The term “HES” can therefore be used to qualify any condition characterized by eosinophilic infiltrates and associated complications, including situations in which the cause of HE is identified (for example, restrictive heart disease occurring in the context of parasitic infections [Löffler's endocarditis]). The categories of HES are further sub-classified according to the pathogenic mechanisms that result in the expansion of eosinophils: primary being the most described myeloproliferative variant in the literature, secondary (reactive) resulting from cytokines that stimulate eosinophilia, or idiopathic being a diagnosis of exclusion (1,4).

The goal of treatment for patients with HES is to reduce the long-term levels of eosinophils in the blood and tissues to reverse and prevent the damage to target organs. Except for patients with imatinib-sensitive HES variants, including those associated with FIP1 like-1 platelet-derived growth factor α-fusion gene (FIP1L1-PDGFRA), the standard of care is the administration of glucocorticoids and cytotoxic/immunosuppressive therapy. However, these drugs have variable efficacy and are often associated with significant morbidity and adverse side effects. The heterogeneous nature of the disease also makes clinical management challenging, with patients typically exhibiting different patterns of disease activity (for example, worsening or relapse of symptoms). Interleukin (IL)-5 is a key regulator of the biology of eosinophils, so therapy directed against the IL-5 pathway has been explored as a potential treatment strategy for patients with HES. Mepolizumab (MEP) is a humanized monoclonal antibody (IgG1, kappa) that acts on human IL-5 with high affinity and specificity (2,3).

## MATERIALS AND METHODS

### Protocol and Registration

The study protocol was registered in the International Prospective Registry of Systematic Reviews (PROSPERO) under the number CRD42021242338. Reporting for this review is in line with the recommendations of the preferred reporting items for systematic reviews and meta-analyses (PRISMA) guidelines.

### Eligibility Criteria

Only those randomized controlled trials (RCTs) and observational studies with a control group, which were published or presented in summary form in English, Spanish, and Portuguese, were included; however, there was no limitation on the year of publication. Studies with the following characteristics were included: (i) Participants: Adult and pediatric patients with HES, fusion of the negative gene for FIP1L1-PDGFRA; (II) Intervention: Use of MEP at any dosage or route of administration; (III) Comparison: Other treatments, including placebo; (IV) Results: Hospitalization rate related to HES, morbidity (new or worsening), relapse/failure, adverse effects related to treatment, prednisone dosage ≤10 mg/day for ≥8 weeks, and eosinophil count <600/μL per ≥8 weeks. The exclusion criteria were as follows: (I) Studies using non-humans and (II) fusion of the positive gene for FIP1L1-PDGFRA.

### Research and Study Selection

The research was exclusively carried out using electronic databases [Medline (https://pubmed.ncbi.nlm.nih.gov/), Cochrane Library (https://www.cochranelibrary.com/), and Lilacs/Bireme (https://lilacs.bvsalud.org/)] from the beginning until March 2021. The sensitive research strategy for the databases consulted was: (Hypereosinophilic Syndrome OR Hypereosinophilic Syndromes OR Eosinophilic Leukemia OR Eosinophilic Leukemias OR Loeffler's Endocarditis OR Loefflers Endocarditis OR Loeffler Endocarditis OR Idiopathic Hypereosinophilic Syndrome OR Idiopathic Hypereosinophilic Syndromes OR Pulmonary Eosinophilia) AND (MEP OR Bosatria OR SB240563 OR Nucala). Two independent researchers screened for eligibility. Any disagreements were resolved by consensus or consultation with a third reviewer.

### Data Collection Process

Two independent reviewers extracted and organized the relevant data in the form of tables. The primary results were prednisone dosage ≤10 mg/day for ≥8 weeks and eosinophil count <600/μL for ≥8 weeks. Secondary results were as follows: Hospitalization rate related to HES, morbidity (new or worsening), relapses/failure, and adverse effects related to treatment.

### Risk of Bias in Individual Studies

The selected evidence was defined as a RCT and submitted to an appropriate critical assessment checklist, covering the following items: randomization, blindfolded allocation, double-blinding, evaluator blinding, losses (<20%), characteristic prognoses, appropriate outcomes, ITT analysis, sample calculation, and early interruption.

### Measures, Summary of Results, and Quality of Evidence

Based on the results of discrete quantitative variables, the differences between their measurements were calculated using the absolute number of events, as well as the sample size of each group. Statistical analyses were performed using the Review Software manager, v.5.4 (RevMan 5.4; Cochrane Collaboration, Oxford, UK), using the risk difference (RD). A 95% confidence interval was adopted, and the level of statistical significance was established with a *p-*value of less than 0.05. Meta-analyses were performed using the fixed-effects model, as they did not show ≥50% heterogeneity. The results of each outcome were graphically analyzed using forest plots. The quality of evidence was analyzed using the Grading of Recommendations Assessment, Development, and Evaluation (GRADE) classification.

## RESULTS

### Study Selection

**Figure 1:**
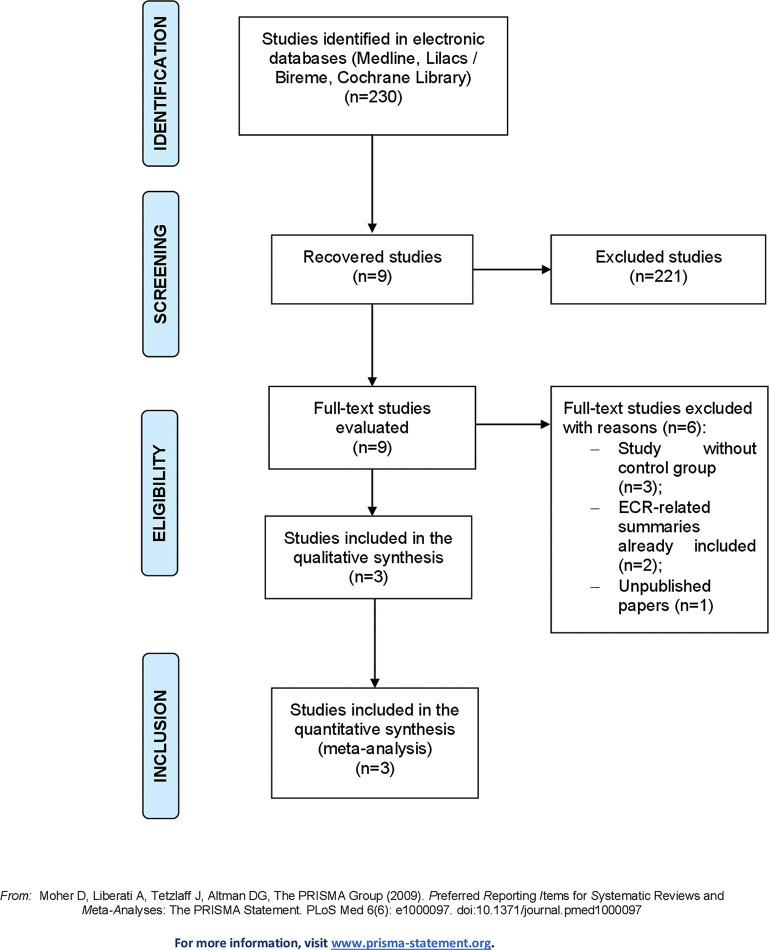
Preferred reporting items for systematic reviews and meta-analyses (PRISMA) diagram detailing the study selection process.

The study selection process adopted, which identified 230 citations in the databases consulted, is demonstrated in Figure 1, and the characteristics of the included studies are summarized in Table 1.

All studies identified were evaluated by title/abstract, and nine studies were selected for full-text review. Of these, three studies had no control group, two were summaries related to an included RCT, and one had not yet been published on the current date. After exclusion, this review included one individual study that was not subjected to meta-analysis and two studies whose characteristics and outcomes could be meta-analyzed.

### Risk of Bias in Studies

In general, the included RCTs had a low risk of bias, as shown in Table 2.

### Results of Individual Studies

The results of the individual studies are summarized in Table 3.

## SUMMARY OF RESULTS

### Relapse/Therapeutic Failure

**Figure 2:**
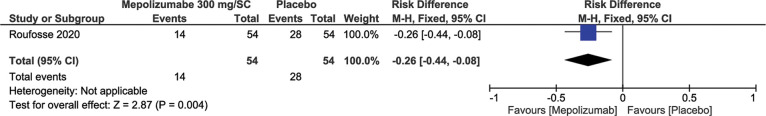
Forest plot reporting the decrease in therapeutic relapse.

**Figure 3:**
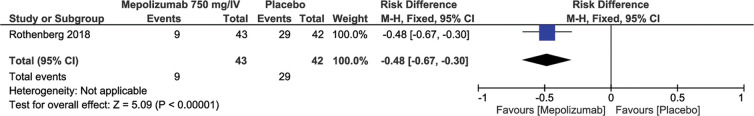
Forest plot reporting the decrease in therapeutic failure.

This outcome was evaluated in two different studies according to the dosage and route of administration of MEP. A study (2) with 108 patients used MEP 300 mg/SC (n=54) and the placebo control group (n=54). For this dosage, it was observed that in the MEP group, there was a 26% reduction in the number of relapses (RD=-0.26; 95% CI: -0.44, -0.08; *p*=0.004) (Figure 2). In another study (3) with 85 patients, 43 patients received MEP at a dosage of 750 mg/IV and 42 patients received the placebo, and the results showed that, in the MEP group, there was a 48% reduction in therapeutic failure (RD=-0.48; 95% CI: -0.67 to -0.30; *p*<0.00001) (Figure 3).

### Treatment-Related Adverse Effects

**Figure 4:**
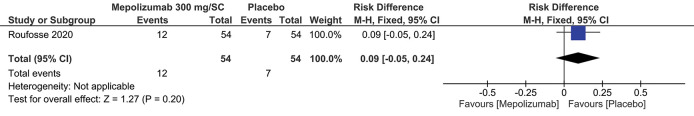
Forest plot study reporting the adverse effects of mepolizumab (MEP) (300 mg/SC].

**Figure 5:**
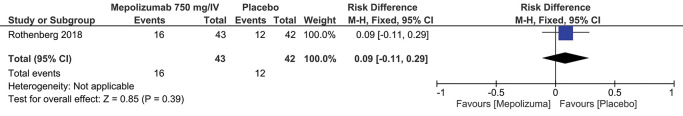
Forest plot reporting the adverse effects of MEP (750 mg/IV].

Similar to the previous outcome, the same studies (2,3) measured the results for this outcome according to the dosage and route of administration. Regardless of the dosage of 300 mg/SC or 750 mg/IV, no statistically significant differences were observed: RD=0.09; 95% CI: -0.05 to 0.24; *p*=0.20 and RD=0.09; 95% CI: -0.11 to 0.29; *p*=0.39, respectively (Figures 4 and 5).

### Prednisone Dosage ≤10 mg/day for ≥8 weeks

**Figure 6:**
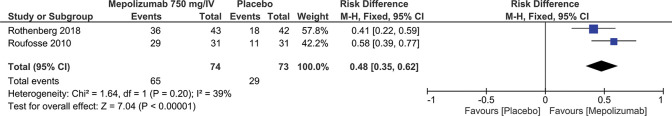
Forest plot reporting the decrease in prednisone dosage.

The decrease in the prednisone dosage in patients considered stable can be measured through the meta-analysis of two studies (3,5) involving 147 patients, of which, 74 patients received MEP at a dosage of 750 mg/IV and 73 patients in the control group received the placebo. The results of the meta-analysis showed that MEP reduces prednisone dosage by up to 48% to ≤10 mg/day for ≥8 weeks (Result: RD=0.48; 95% CI: 0.35 to 0.62; *p*<0.00001) (Figure 6).

### Eosinophil Count <600/μL for ≥8 weeks

**Figure 7:**
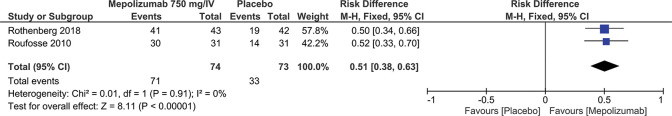
Forest plot reporting the decrease in eosinophil count.

Similar to the previous outcome, the same studies (3,5) also evaluated the decrease in circulating eosinophils in stable patients. In this case, the meta-analysis showed a 51% reduction in the eosinophil count to <600/μL for ≥8 weeks (RD=0.51; 95% CI: 0.38 to 0.63; *p*<0.00001) (Figure 7).

These outcomes provide a moderate degree of evidence according to the GRADE Working Group (Table 4).

## DISCUSSION

MEP inhibits the binding of IL-5 to the α chain of the IL-5 receptor expressed in eosinophils, which allows its use in HES, more specifically in secondary and lymphocytic cells. These syndromes are associated with excessive production of cytokines, such as IL-3, IL-5, and the macrophage granulocyte colony-stimulating factor (GM-CSF), which in turn promote the maturation and survival of eosinophils, while inhibiting their apoptosis. IL-5 is the most important factor for this process; however, its binding and action can be inhibited by the use of anti-IL-5 to reduce eosinophilia (6).

Unlike MEP for the management of HES, large studies are available for patients with severe eosinophilic asthma, including RCTs and systematic reviews (7-15). For HES, there are still not many studies, mainly RCTs. Most studies that we found were reports and case series, which do not allow an in-depth assessment of the topic. Despite this review strictly following the criteria imposed by the PRISMA statement and selecting studies with adequate methodological quality, it was not possible to eliminate some limitations. The included studies had a small number of patients and generally belonged to the same group of authors. In addition, the studies used different inclusion criteria, dosages, and routes of administration, making it difficult to carry out a meta-analysis. Perhaps a large part of these limitations is inherent to the low prevalence of the disease, as well as the criteria for accurate diagnosis.

The other outcomes listed as secondary (hospitalization rate and morbidity) were not suitable for synthesis. The only study available that categorically assessed these outcomes has been published in the manuscript form (16).

The use of MEP as a treatment for hypereosinophilic syndromes is an extremely relevant issue for immunology, especially when there is an excessive increase in cytokines causing eosinophilia. In addition, it presents a possible therapeutic hope with anti-IL-5, which, compared to placebo, decreased the eosinophil count in controlled patients.

This systematic review reveals a scarcity of studies with adequate scientific rigor, especially RCTs. This scarcity prevents an adequate assessment of the use of MEP at different dosages and routes of administration for the treatment of HES, both for the uncontrolled and clinically controlled patients. However, MEP, when compared to placebo, seems to be superior for the following outcomes: decreased therapeutic failure, prednisone dose, and eosinophil count in controlled patients.

Due to the present lack of evidence, it is not yet possible to draw definitive conclusions about the significance of its use.

## CONCLUSION

Despite the positive effects observed regarding the decrease in the prednisone dosage and eosinophil count in controlled patients as well as the decrease in relapses/therapeutic failures, these results should be interpreted with caution. The lack of robust evidence inherent in the available studies creates ambiguity regarding the significance of this benefit.

## AUTHOR CONTRIBUTIONS

Alves Júnior JM made considerable contributions to the design and postulation of the study, the definition of technical content, literature search, data analysis, statistical analysis, manuscript preparation, drafting, writing, critical review, and approval of the manuscript final version for publication. Prota FE and Villagelin D were involved in the data analysis, statistical analysis, manuscript preparation, writing, drafting, critical review for important intellectual content, and approval of the manuscript final version for publication. Bernardo WM and Bley F provided support for the entire process of developing and reviewing this systematic review.

## Figures and Tables

**Table 1:** Characteristics of the studies included in the meta-analysis.

DESCRIPTION OF THE STUDIES						
STUDIES	DESIGN	POPULATION	INTERVENTION	CONTROL	OUTCOMES	FOLLOW-UP TIME
Roufosse et al. 2020 (2)	RCT	Patients≥12, negative diagnosis of FIP1L1-PDGFRA, uncontrolled HES (AEC≥1000 cell/μL or ≥2 relapses in the last 12 months) and who were receiving previous therapy. (n=108)	MEP 300 mg/SC (n=54)	Placebo (n=54)	Relapses/failure and treatment-related adverse events	32 weeks
Roufosse et al. 2010 (5)	RCT	Patients≥18 years old with a diagnosis of HES, stable (prednisone 20-60 mg/day), with no new clinical sign or worsening, negative diagnosis of FIP1L1-PDGFRA and AEC≤1000 cell/μL (n=62)	MEP 750 mg/IV (n=31)	Placebo (n=31)	Prednisone dosage ≤10 mg/day for ≥8 weeks and eosinophil count <600/μL for ≥8 weeks	36 weeks
Rothenberg et al. 2008 (3)	RCT	Patients≥18 years old with a diagnosis of HES, stable (prednisone 20-60 mg/day), without new clinical sign or worsening, negative diagnosis of FIP1L1-PDGFRA and AEC≤1000 cell/μL. (n=85)	MEP 750 mg/IV (n=43)	Placebo (n=42)	Prednisone dosage ≤10 mg/day for ≥8 weeks, eosinophil count <600/μL for ≥8 weeks, relapse/failure rate	36 weeks

**Table 2:** Global risk of bias in each study.

RISK OF BIAS										
STUDIES	RANDOMIZATION	BLIDFOLDED ALLOCATION	DOUBLE BLINDING	EVALUATOR BLINDING	LOSSES (<20%)	PROGNOSTIC CHARACTERISTICS	APPROPRIATE OUTCOMES	ITT ANALYSIS	SAMPLE CALCULATION	EARLY INTERRUPTION
Roufosse et al. 2020 (2)										
Roufosse et al. 2010 (5)										
Rothenberg et al. 2008 (3)										

**Table 3:** Summary of results according to the outcome of each study.

STUDIES	RESULTS											
	Related hospitalization rate		New morbidity/worsening		Relapses that require therapeutic change		Adverse effects related to the agent		Prednisone dosage ≤10 mg/day for ≥8 weeks		Eosinophil count <600/μL for ≥8 weeks	
	MEP	CONTROL	MEP	CONTROL	MEP	CONTROL	MEP	CONTROL	MEP	CONTROL	MEP	CONTROL
Roufosse et al. 2020 (2)					14 de 54 (26%)	28 de 54 (52%)	12 de 54 (22%)	7 de 54 (13%)				
Roufosse et al. 2010 (5)									29 de 31 (93,5%)	11 de 31 (35,5%)	30 de 31 (97%)	14 de 31 (45%)
Rothenberg et al. 2018 (3)					9 de 43 (21%)	29 de 42 (69%)	16 de 43 (37%)	12 de 42 (29%)	36 de 43 (84%)	18 de 42 (43%)	41 de 43 (95%)	19 de 42 (45%)

**Table 4:** Grading of Recommendations Assessment, Development, and Evaluation (GRADE) evaluation.

Certainty assessment							No. of patients		Effect		Certainty	Importance
No. of studies	Study design	Risk of bias	Inconsistency	Indirect evidence	Inaccuracy	Other considerations	Mepolizumab 750 mg/IV	Placebo	Relative (95% CI)	Absolute (95% CI)		
**Prednisone dosage ≤10 mg/day for ≥8 weeks**												
2	randomized clinical trials	serious^a^	not serious	not serious	not serious	none	59/67 (88.1%)	28/67 (41.8%)	not estimable	460 less per 1.000 (from 600 less to 320 less)	⨁⨁⨁◯ MODERATE	
**Eosinophil count ≤600/μL for ≥8 weeks**												
2	randomized clinical trials	serious^a^	not serious	not serious	not serious	none	65/67 (97.0%)	32/67 (47.8%)	not estimable	490 less per 1.000 (from 620 less to 360 less)	⨁⨁⨁◯ MODERATE	

Explanations

CI: Confidence interval.

a. Losses >20%.
